# Glucagonlike Peptide-1 (GLP-1) Receptor Agonists for Cardiometabolic Risk in Severe Mental Illness: A Narrative Review

**DOI:** 10.7759/cureus.110038

**Published:** 2026-06-01

**Authors:** Catarina Domingues, Maria Ferreira, Rosa Pinho

**Affiliations:** 1 Family Medicine, USF Vale do Vouga, Unidade Local de Saúde de Entre Douro e Vouga, São João da Madeira, PRT; 2 Psychiatry, Unidade Local de Saúde de Entre Douro e Vouga, Santa Maria da Feira, PRT; 3 Family Medicine, USF Vale do Vouga, Unidade Local de Saúde de Entre Douro e Vouga, Oliveira de Azeméis, PRT

**Keywords:** “antipsychotic agents”, “bipolar disorder”, cardiometabolic risk, “glucagon-like peptide 1 receptor agonists”, “schizophrenia”

## Abstract

People with severe mental illness face a substantially increased cardiometabolic risk and premature mortality, largely driven by antipsychotic-associated metabolic disturbances, including significant weight gain, insulin resistance, dyslipidemia, impaired glucose homeostasis, and increased rates of metabolic syndrome. Despite existing recommendations for monitoring and early intervention, managing this risk remains a significant clinical challenge. Glucagonlike peptide-1 receptor agonists have emerged as a potential adjunctive therapeutic strategy in this context.

This narrative review evaluated the efficacy and safety of these agents in adults with severe mental illness receiving chronic antipsychotic treatment. A structured search was conducted in PubMed/MEDLINE and the Cochrane Library, complemented by relevant clinical guidelines and consensus documents. Randomized controlled trials, systematic reviews, and meta-analyses published between 2015 and 2025 in English or Portuguese were included, and the strength of evidence was assessed using the Strength of Recommendation Taxonomy.

Nine studies were included, comprising six randomized controlled trials and three evidence synthesis studies. Liraglutide and semaglutide consistently demonstrated clinically meaningful reductions in body weight and improvements in glycemic control, without evidence of worsening psychiatric symptoms. In contrast, exenatide did not show additional weight loss compared with placebo, although some hemodynamic benefits were observed. The most common adverse events were mild to moderate gastrointestinal symptoms. Overall, the available evidence suggests a favorable balance between cardiometabolic benefits and psychiatric safety, particularly in patients treated with high metabolic risk antipsychotics such as clozapine and olanzapine.

GLP-1 receptor agonists appear to be a promising adjunctive option for cardiometabolic risk reduction in this population. These findings support their consideration as a clinically relevant strategy, especially in patients at higher cardiometabolic risk. However, current evidence is limited by small sample sizes and short follow-up periods, and further long-term studies are needed to support broader clinical implementation.

## Introduction and background

Patients with severe mental illness, including schizophrenia, bipolar disorder, and schizoaffective disorder, have a higher burden of physical illness and increased rates of premature mortality. Cardiovascular disease remains one of the leading causes of death in this population [1-3]. This excess risk is partly explained by modifiable factors such as smoking, physical inactivity, dietary habits, and disparities in access to healthcare. However, it is also closely linked to the cardiometabolic effects of antipsychotic treatment. In particular, several second-generation antipsychotics have been consistently associated with weight gain, disturbances in glucose metabolism, and dyslipidemia, all of which contribute to the development of metabolic syndrome and increased cardiovascular risk [2,4,5]. Metabolic syndrome affects a substantial proportion of individuals with severe mental illness, with prevalence estimates frequently exceeding 30% and reaching over 50% in some populations, substantially higher than rates observed in the general population [1,4].

Given this increased vulnerability, several clinical recommendations highlight the importance of baseline assessment and regular monitoring of physical health parameters during antipsychotic treatment. In bipolar disorder, the National Institute for Health and Care Excellence recommends assessing weight or body mass index, pulse, blood pressure, fasting glucose or glycated hemoglobin, and lipid profile before initiating treatment, with ongoing follow-up over time [6]. Similar recommendations exist for psychosis and schizophrenia, including the assessment of diabetes risk and periodic monitoring of metabolic parameters [7]. In parallel, consensus statements from scientific societies such as the British Association for Psychopharmacology have emphasized the need to actively address weight gain and metabolic disturbances associated with psychosis and antipsychotic use. In this context, regular monitoring and early intervention are considered key strategies to limit progression to diabetes and cardiovascular disease [4,8].

Despite these recommendations, recent studies suggest that metabolic monitoring in routine clinical practice often falls short of what is advised. This gap has been associated with delays in recognizing and managing cardiometabolic abnormalities [1,3]. Several initiatives have therefore emerged to improve care integration and to standardize the assessment of physical health in people with severe mental illness [3,9].

Traditional pharmacological approaches for weight management in patients with severe mental illness remain challenging and have important limitations, highlighting the need for alternative therapeutic strategies [4]. Consequently, and given the limitations of lifestyle interventions when used in isolation, there has been growing interest in glucagonlike peptide-1 (GLP-1) receptor agonists as an adjunctive treatment approach. This interest is particularly relevant for patients treated with antipsychotics associated with higher metabolic risk, such as clozapine and olanzapine [4,5,8].

Although GLP-1 receptor agonists are not yet specifically incorporated into major psychiatric treatment guidelines, emerging evidence points toward consistent benefits in weight reduction and improvements in metabolic parameters. The primary objective of this narrative review was to evaluate the available evidence regarding the efficacy and safety of GLP-1 receptor agonists as an adjunctive therapeutic strategy for reducing cardiometabolic risk in adults with severe mental illness receiving chronic antipsychotic treatment compared with placebo or usual care. Secondary objectives included assessing their effects on body weight, body mass index, waist circumference, and glycemic control; evaluating tolerability and psychiatric safety; identifying patient subgroups more likely to benefit, particularly those receiving antipsychotics associated with higher metabolic risk, especially clozapine and olanzapine; and examining limitations in the available evidence, knowledge gaps, and implications for future research and clinical practice.

## Review

Methods

This study was conducted as a narrative review focused on a specific clinical question, structured according to the patient, intervention, comparison, and outcome (PICO) framework. The review aimed to summarize and critically appraise the available evidence regarding the role of GLP-1 receptor agonists in reducing cardiometabolic risk among individuals with severe mental illness. The strength of evidence was assessed using the Strength of Recommendation Taxonomy (SORT). As this study was conducted as a narrative review rather than a systematic review, a formal risk-of-bias assessment tool was not applied. Instead, methodological quality was considered through study design characteristics and overall appraisal of evidence strength.

A structured literature search was performed using PubMed/MEDLINE and the Cochrane Library. This was complemented by a targeted review of relevant clinical guidelines and consensus documents, including those from the National Institute for Health and Care Excellence, the British Association for Psychopharmacology, the World Health Organization, and National Health Service (NHS) England.

The search strategy included combinations of Medical Subject Headings (MeSH) terms and keywords, including “schizophrenia,” “bipolar disorder,” “schizoaffective disorder,” “antipsychotic agents,” and “glucagonlike peptide-1 receptor agonists,” combined using Boolean operators (AND/OR). Search terms were adapted according to database requirements. Eligible studies included randomized controlled trials, systematic reviews, and meta-analyses published between 2015 and 2025 in English or Portuguese, with full-text availability.

Inclusion criteria were defined according to the PICO framework. The population included adults (≥18 years) with severe mental illness, specifically schizophrenia, bipolar disorder, or schizoaffective disorder, receiving antipsychotic treatment regardless of generation, including agents associated with higher metabolic risk. The intervention consisted of GLP-1 receptor agonists used as adjunctive pharmacological therapy, such as liraglutide or semaglutide. Comparators included placebo or usual care, with or without lifestyle interventions. Outcomes of interest included cardiometabolic parameters, including body weight, body mass index, waist circumference, and glycemic control (HbA1c or fasting glucose), as well as safety and tolerability outcomes, including adverse events and treatment discontinuation.

Studies were excluded if they involved populations without severe mental illness or exclusively pediatric populations, preclinical studies, or if GLP-1 receptor agonists were not the primary intervention or their effects could not be clearly isolated. Studies focusing solely on outcomes not directly relevant to cardiometabolic risk were also excluded, as were opinion articles, editorials, letters to the editor, conference abstracts, and publications without full-text availability, or those published outside the defined time frame or in languages other than English or Portuguese.

Study selection was performed according to predefined inclusion and exclusion criteria, with full-text assessment of eligible articles. Any uncertainties regarding study eligibility were resolved through re-evaluation of predefined criteria and repeated review until consensus was reached. As this review followed a narrative design, no quantitative synthesis, meta-analysis, or inferential statistical analysis was performed.

Results

The literature search identified 12 potentially eligible studies. In parallel, a targeted search of clinical guidelines and international consensus documents was also performed, including those from the National Institute for Health and Care Excellence, the British Association for Psychopharmacology, the World Health Organization, and NHS England [3,4,6-9]. No duplicate publications were identified. Two studies were excluded following full-text review: one focused exclusively on cognitive outcomes and another used metformin as the primary intervention, not meeting the predefined inclusion criteria. In addition, some publications represented follow-up analyses or secondary reports derived from the same randomized controlled trial and were therefore considered complementary reports rather than independent studies. Consequently, nine studies were included in the final synthesis, comprising six randomized controlled trials and three evidence synthesis studies, including systematic reviews and meta-analyses. Across randomized controlled trials, sample sizes ranged from 45 to 154 participants and primarily included adults with schizophrenia or schizophrenia spectrum disorders receiving antipsychotic treatment, with variable demographic characteristics across studies.

Clinical guidelines and consensus statements consistently emphasized the importance of monitoring and managing cardiometabolic risk in patients with severe mental illness treated with antipsychotics. However, none provided specific recommendations regarding the use of GLP-1 receptor agonists in this context. These sources were therefore not included as direct evidence in the synthesis of the results.

Of these, six were placebo-controlled randomized trials evaluating GLP-1 receptor agonists as adjunctive therapy in adults with severe mental illness receiving antipsychotic treatment [10-15]. The interventions included weekly semaglutide (up to 1.0 mg) [10,11], daily liraglutide (up to 1.8 mg) [12-14], and once-weekly extended-release exenatide (2 mg) [15]. Study duration ranged from 16 to 30 weeks [10-12,15], with one trial including an additional one-year observational follow-up after the randomized phase [13].

Primary outcomes focused mainly on cardiometabolic parameters, particularly glycemic control (HbA1c and glucose tolerance) and body weight [10-12]. Secondary outcomes included body mass index, waist circumference, body composition, lipid profile, and cardiovascular parameters, as well as psychiatric safety assessed through symptom scales, quality of life measures, and reporting of serious adverse events [10-15].

Overall, studies evaluating semaglutide and liraglutide demonstrated clinically meaningful reductions in body weight and consistent improvements in glycemic control, with no evidence of worsening psychiatric symptoms [10-14]. In contrast, the trial involving exenatide did not show additional weight loss compared with placebo, although it was associated with hemodynamic benefits, including improvements in blood pressure and arterial stiffness, as reflected by a reduction in pulse wave velocity [15].

Regarding safety, the most frequently reported adverse events were gastrointestinal and were generally mild to moderate and transient [10-15]. No consistent differences were observed between groups in the incidence of serious psychiatric events, suggesting no relevant negative impact on clinical stability [10-15]. In addition, one substudy did not identify significant changes in bone remodelling markers following 16 weeks of liraglutide treatment [14]. A summary of the main characteristics and findings of the included studies is presented in Table 1.

**Table 1:** Summary of clinical studies evaluating GLP-1 receptor agonists in patients with severe mental illness receiving antipsychotic treatment HbA1c: glycated hemoglobin; BMI: body mass index; HDL: high-density lipoprotein; LDL: low-density lipoprotein; BP: blood pressure; OGTT: oral glucose tolerance test; PANSS: Positive and Negative Syndrome Scale; CGI-S: Clinical Global Impression-Severity; QoL: quality of life; CTX: C-terminal telopeptide; P1NP: procollagen type 1 N-terminal propeptide; RCT: randomized controlled trial Data are presented as reported in the included studies

Author (year)	Study design	Population	Intervention (dose)	Duration	Primary outcome	Main cardiometabolic results	Psychiatric safety	Level of evidence
Ganeshalingam et al., 2025 [10]	RCT	Schizophrenia; second-generation antipsychotics; prediabetes; BMI ≥ 27 (n = 154)	Semaglutide s.c. 1 mg/week vs placebo	30 weeks	Δ HbA1c	HbA1c −0.46%; weight −9.2 kg; ↑ HDL; ↓ triglycerides	No worsening in PANSS-6 or mental QoL	2
Sass et al., 2025 [11]	RCT	Schizophrenia spectrum; clozapine/olanzapine ≤5 years; dysglycemia (n = 73)	Semaglutide s.c. 1 mg/week vs placebo	26 weeks	Δ HbA1c	HbA1c −0.25%; weight −9.2 kg; waist −7.0 cm; ↓ fat mass	No differences in PANSS-6, CGI-S or QoL	2
Larsen et al., 2017 [12]	RCT	Schizophrenia; clozapine/olanzapine; BMI ≥ 27; prediabetes (n = 103)	Liraglutide s.c. 1.8 mg/day vs placebo	16 weeks	Glucose tolerance (OGTT)	OGTT normalization in 64%; weight −5.3 kg; ↓ waist circumference; ↓ LDL; ↓ systolic BP	No clinical worsening; fewer serious adverse events observed	2
Maagensen et al., 2021 [14]	RCT (substudy)	Subgroup of the Larsen trial (n = 78)	Liraglutide s.c. 1.8 mg/day vs placebo	16 weeks	Bone turnover markers (CTX, P1NP)	No significant changes in bone turnover markers	No new safety signals	3
Ishøy et al., 2017 [15]	RCT	Schizophrenia spectrum; BMI ≥ 30; various antipsychotics (n = 45)	Exenatide extended-release 2 mg/week vs placebo	12-16 weeks	Weight change	Similar weight loss in both groups; ↓ systolic BP; ↓ arterial stiffness	No clinically relevant psychiatric changes	2
Svensson et al., follow-up [13]	Observational follow-up study (extension of RCT)	Participants from the 2017 trial (n = 103)	Liraglutide continued vs discontinued (observational)	1 year	Weight maintenance	Partial maintenance of weight loss and glycemic control when treatment continued	Safety profile similar to randomized phase	4

Three evidence synthesis studies were included: an individual participant data meta-analysis, a systematic review, and a network meta-analysis, evaluating pharmacological strategies targeting cardiometabolic risk associated with antipsychotic treatment in patients with schizophrenia or schizophrenia spectrum disorders [16-18]. These studies are summarized in Table 2.

**Table 2:** High-level evidence on pharmacological strategies for antipsychotic-associated cardiometabolic risk in severe mental illness BMI: body mass index; HbA1c: glycated hemoglobin; DXA: dual-energy X-ray absorptiometry; RCT: randomized controlled trial Results are presented as changes compared with control groups unless otherwise specified. Arrows indicate direction of change (↑ increase; ↓ decrease). GLP-1 receptor agonists include liraglutide and exenatide

Author (year)	Study design	Population	Intervention	Duration	Outcomes assessed	Main cardiometabolic results	Psychiatric safety/notes	Level of evidence
Siskind et al., 2019 [16]	Individual participant data (IPD) meta-analysis	Adults with schizophrenia spectrum disorders; overweight/obese; on antipsychotics (n=168)	Exenatide 2 mg/week or liraglutide up to 1.8 mg/day vs control	12-24 weeks (mean 16.2)	Weight, BMI, waist circumference, HbA1c, glucose, lipid profile, DXA, blood pressure	Associated with −3.71 kg vs control; ↓ BMI; ↓ waist circumference; ↓ HbA1c (~0.3%); greater effect in patients treated with clozapine or olanzapine	Frequent nausea; no evidence of worsening psychotic symptoms	1
Lee et al., 2022 [17]	Systematic review	Adults with schizophrenia/psychosis on antipsychotics (4 RCTs)	Liraglutide, semaglutide, naltrexone-bupropion, others	Studies published between 2000 and 2022 (duration 16-52 weeks)	Weight, BMI, waist circumference, HbA1c, lipid profile, blood pressure	Liraglutide showed the strongest evidence (−5.3 kg); limited data for semaglutide	Overall psychiatric stability; frequent gastrointestinal adverse events	3
Hegde et al., 2024 [18]	Network meta-analysis	Trials in schizophrenia with antipsychotic-induced weight gain (68 RCTs)	Metformin, topiramate, liraglutide, others	Variable	Body weight	Liraglutide was associated with −5.2 kg weight reduction; a similar effect to metformin/topiramate	Limited psychiatric safety data; with a primary focus on weight outcomes	1

The individual participant data meta-analysis by Siskind et al. pooled data from three randomized controlled trials evaluating GLP-1 receptor agonists, namely, weekly exenatide and daily liraglutide, including 168 participants. The analysis focused on clinically relevant cardiometabolic outcomes, including body weight, body mass index, waist circumference, and glycemic control, while also incorporating a structured assessment of safety [16].

Overall, GLP-1 receptor agonists were associated with an additional mean weight reduction of 3.7 kg compared with control, alongside significant reductions in waist circumference, body mass index, and glycemic parameters. These effects appeared more pronounced in patients treated with clozapine or olanzapine. Adverse events were predominantly gastrointestinal, particularly nausea, with no evidence of worsening psychotic symptoms. However, heterogeneity in the assessment tools used across studies limited the quantitative evaluation of psychiatric safety [16].

The systematic review by Lee et al. examined pharmacological treatments approved for antipsychotic-associated weight gain, including liraglutide, semaglutide, and the combination of naltrexone-bupropion. Among these, liraglutide showed the most consistent evidence, being associated with clinically meaningful reductions in body weight, body mass index, and waist circumference, as well as improvements in glycemic control. In contrast, evidence for semaglutide was mainly derived from small observational studies, while naltrexone-bupropion did not demonstrate consistent benefit in randomized controlled trials [17].

The network meta-analysis by Hegde et al. provided an indirect comparison of multiple pharmacological interventions used in this setting. Liraglutide at a dose of 1.8 mg was associated with weight loss of a magnitude comparable to that observed with metformin and topiramate, although supported by a more limited evidence base in the case of GLP-1 receptor agonists. Data on psychiatric safety and cardiometabolic outcomes beyond weight were limited in this analysis [18].

Discussion

This review examined the available evidence regarding the efficacy and safety of GLP-1 receptor agonists as adjunctive therapy for cardiometabolic risk reduction in adults with severe mental illness receiving chronic antipsychotic treatment. Across the available studies, findings from randomized controlled trials and evidence synthesis studies consistently indicate a clinically meaningful benefit, particularly for liraglutide and semaglutide, mainly in terms of weight reduction and improved glycemic control when compared with placebo or usual care [10-14,16,17]. These effects were observed across key cardiometabolic parameters, including body weight, body mass index, waist circumference, and HbA1c, all of which are directly linked to cardiovascular risk in this population [10-18].

The magnitude of weight loss reported in trials with liraglutide and semaglutide, typically ranging between 5 kg and 9 kg over relatively short periods (16 to 30 weeks) [10-13], exceeds what is usually achieved with lifestyle interventions alone in patients with severe mental illness. These findings further support the potential clinical relevance of GLP-1 receptor agonists in this population. Beyond weight reduction, improvements in glycemic control, including reversal of prediabetes in some cases, further support the role of GLP-1 receptor agonists as a clinically relevant cardiometabolic strategy [10-13,16]. This is especially relevant given the high and often underrecognized burden of type 2 diabetes in this population [1,2]. The mechanisms underlying these benefits are likely multifactorial. GLP-1 receptor agonists promote weight reduction primarily through delayed gastric emptying, enhanced satiety, and reduced caloric intake mediated by central appetite regulation pathways. Improvements in glycemic control result from glucose-dependent stimulation of insulin secretion and suppression of glucagon release [16-18]. Additional favorable effects on lipid metabolism and cardiometabolic profiles have also been described in evidence synthesis studies, potentially contributing to reductions in overall cardiovascular risk [16-18].

Regarding safety and tolerability, the available evidence suggests an overall favorable profile of GLP-1 receptor agonists in patients with severe mental illness. Reported adverse events were predominantly gastrointestinal, particularly nausea, vomiting, diarrhea, and constipation and were generally mild to moderate in severity and transient [10-15]. No consistent evidence of worsening psychiatric symptoms, increased psychiatric admissions, or clinical destabilization was observed [10-18]. In addition, more recent data did not show clinically meaningful changes in clozapine plasma levels, helping to mitigate concerns about potential pharmacokinetic interactions in a particularly vulnerable subgroup [11].

The analysis also suggests that the benefits of GLP-1 receptor agonists may be more pronounced in patients treated with antipsychotics associated with higher metabolic risk, particularly clozapine and olanzapine [11,12,16]. This finding is clinically relevant, as switching these agents is often not feasible, especially in treatment-resistant schizophrenia [8]. In this context, GLP-1 receptor agonists may help address a clinically relevant therapeutic gap, offering an adjunctive strategy to mitigate cardiometabolic burden without compromising psychiatric stability, based on current evidence.

However, these findings should be interpreted with caution. Most available trials include relatively small sample sizes and short follow-up periods, and there is limited evidence regarding the long-term sustainability of benefits, particularly after treatment discontinuation [10-18]. Furthermore, methodological heterogeneity across studies, including differences in inclusion criteria, antipsychotic regimens, and outcome definitions, limits direct comparability between studies and interpretation of overall findings [16-18].

In evidence synthesis studies, the assessment of psychiatric safety remains particularly challenging, largely due to variability in the scales used and the absence of standardized psychiatric endpoints in some trials [16]. Most of the available data focus on patients with schizophrenia or schizophrenia spectrum disorders, with limited evidence specifically addressing bipolar or schizoaffective disorder [10-17].

This review also has limitations. As a narrative review, it was not conducted using formal systematic review methodology and did not include quantitative synthesis or formal risk-of-bias assessment. In addition, only studies published in English or Portuguese were included, potentially limiting the inclusion of relevant evidence from other sources. Further research is needed to clarify the role of GLP-1 receptor agonists across different diagnostic groups and to inform future clinical recommendations.

## Conclusions

Current evidence suggests that GLP-1 receptor agonists may represent an effective adjunctive option for cardiometabolic risk reduction in adults with severe mental illness receiving antipsychotic treatment, with particular relevance in patients treated with clozapine or olanzapine. The observed benefits, especially in terms of weight reduction and glycemic control, were achieved without consistent evidence of worsening psychiatric symptoms, supporting their potential role in clinical practice. Although not yet formally incorporated into current guidelines, the available data support consideration of GLP-1 receptor agonists in selected patients, particularly those at higher cardiometabolic risk, with appropriate clinical monitoring. However, uncertainties remain regarding the long-term sustainability of these effects. Further studies with larger sample sizes and longer follow-up are needed to clarify their role and support their integration into clinical recommendations.
